# Assisted Reproductive Technologies and Offspring's School Grades by Maternal Education

**DOI:** 10.1111/ppe.70181

**Published:** 2026-07-16

**Authors:** Alice Goisis, Øystein Kravdal

**Affiliations:** ^1^ Centre for Longitudinal Studies, Social Research Institute University College London London UK; ^2^ Centre for Fertility and Health Norwegian Institute of Public Health Oslo Norway

**Keywords:** assisted reproductive technologies, mother's education, Norway, school scores

## Abstract

**Background:**

Children conceived through assisted reproductive technologies (ART) often achieve higher school grades than spontaneously conceived peers in unadjusted analyses, a pattern largely attributed to the socio‐economic advantage of parents who use ART. However, as ART is increasingly utilised by less advantaged groups, it is important to understand how ART‐conceived children fare when their parents have lower levels of education.

**Objective:**

The objective of this study is to examine whether the association between conception through assisted reproductive technologies and school grades at age 16 differs by maternal education.

**Methods:**

We conducted a population‐based cohort study of all live‐born children in Norway from 1985 to 2002 who completed compulsory schooling (*n* = 929,017), using nationwide register data. The exposure was conception through ART (1.0%), and the outcome was the standardised grade point average (GPA) at age 16. Analyses were stratified by maternal education (primary/secondary vs. tertiary). Using linear regression models, we estimated differences in GPA between ART‐ and spontaneously conceived children. We progressively adjusted for child sex, birth cohort, plurality, birth order, maternal age, parental income and family structure.

**Results:**

Among children of lower‐educated mothers, mean GPA was higher for ART‐conceived than spontaneously conceived children in baseline models (0.17, 95% confidence interval [CI] 0.14, 0.19). Among children of tertiary‐educated mothers, the corresponding difference was 0.06 (95% CI 0.03, 0.09). After adjustment, the differences were slightly negative but small in magnitude in both education groups (primary/secondary: −0.18, 95% CI −0.21, −0.16; tertiary: −0.08, 95% CI −0.11, −0.05).

**Conclusions:**

Both ART‐conceived children of less‐ and more‐educated mothers appear advantaged in unadjusted analyses. After accounting for parental characteristics, differences were small in both education groups, reflecting socio‐demographic selection into treatment within each group. These findings suggest that as ART use expands across more socio‐economically diverse populations, it is unlikely to independently improve or worsen children's school performance across educational strata.

## 1 Background

The last decades have witnessed a remarkable increase in the use of Assisted Reproductive Technologies (ART). In some countries, such as Denmark and Spain, 9% of the newborn children are now conceived via ART, and since this technique became available, it has resulted in over 10 million births worldwide [1]. This trend has generated scientific interest in the longer‐term development and well‐being of ART‐conceived children, such as their school outcomes [2, 3, 4]. Previous studies show that children conceived via ART have, when looking at unadjusted models, higher school grades than children conceived spontaneously [5, 6, 7]. They argue that this is due to selection into seeking ART to conceive since couples who undergo ART are, on average, socio‐economically advantaged [8, 9, 10]. Indeed, when models adjust for parental socio‐demographic characteristics, the positive association between being conceived via ART and school grades is attenuated. Control for parental education appears to be particularly important [5, 11, 12]. In some studies, the adjusted association becomes negative, but it remains small in magnitude.

These findings raise important questions about how ART‐conceived children fare when their parents have lower levels of education. On the one hand, if the apparent ART advantage reflects the socio‐demographic resources of parents who undergo these treatments, then children of less educated parents—who typically have fewer resources—may not experience the same compensatory benefits, resulting in a smaller or even negative unadjusted association between ART and school performance. On the other hand, within the lower‐educated group, couples who conceive via ART might also be socio‐economically selected in ways that buffer their children's outcomes, leading to a stronger unconditional association between ART and school performance. It is therefore difficult to know a priori whether the relative advantage of ART‐conceived children, and the mechanisms that explain it, differ across education groups. In addition, even after accounting for parental socio‐demographic characteristics, the net association between ART and children's school performance may differ by parental education if the returns to family resources—or the unobserved factors linked to ART and infertility—vary across educational groups. Addressing these knowledge gaps is important, as ART treatments are increasingly utilised by more socially diverse and less advantaged groups [13].

The aims of this study are two‐fold. First, we conduct education‐specific analyses to test whether ART‐conceived children's school grades differ from those of their spontaneously conceived peers before adjustment for parents' socio‐demographic characteristics, to document how ART‐conceived children of lower and higher‐educated mothers are doing compared to their peers. Our second aim is to test whether the factors explaining the ART–school grades association differ between children of lower and higher‐educated mothers. ART‐conceived children are typically first‐born, have older mothers, and have parents with relatively high incomes who are particularly likely to live together. These factors should contribute positively to ART children's school performance [14], so by controlling for them, we expect to see a more negative association between being ART‐conceived and school grades. However, the factors linking ART to children's school outcomes may vary by the mother's education, as children of less educated mothers may not benefit from these additional advantages and resources. Taken together, the analyses test whether the unconditional and adjusted ART–school performance associations vary by maternal education and explore the factors underlying these associations in higher‐ and lower‐educated groups.

We focus on Norway, where ART treatments are heavily subsidised and where, although more advantaged couples are consistently more likely to give birth after ART, the educational gradient has closed across birth cohorts [8]. In addition, Norway has a unique population register, which enables us to conduct this research with a large sample of ART‐conceived children and nationally representative data.

## 2 Methods

### 2.1 Data Sources

This study utilises Norwegian register data: the Population Register, the Medical Birth Register, and national databases on education and income. We included all live births in Norway between 1985 and 2002. The first registered ART delivery in Norway was in 1984, but we only have school scores from the 1985 cohort onwards. Our information on sociodemographic characteristics ends in 2018, when the 2002 cohort attained age 16. Personal identifiers of the child, the mother, and the father were available based on unique national identification numbers. Our sample consisted of 929,017 live‐born children, of whom 9100 (1%) were ART‐conceived.

### 2.2 Exposure: Assisted Reproductive Technology

The exposure variable is whether the child was conceived through Assisted Reproductive Technology (ART) or spontaneously, as recorded in the Medical Birth Register. We identified all children in Norway who were registered as being born after ART (in vitro fertilisation (IVF) or intracytoplasmic sperm injection (ICSI) or registered with an unknown procedure) from the Medical Birth Register. The data do not provide information on intrauterine insemination (IUI) cycles. In the public clinics in Norway, access to ART services is subsidised through the national health plan, which covers up to three ART cycles and the transfer of all fresh and thawed embryos resulting from these cycles.

### 2.3 Outcome: School Grades at Age 16

The outcome variable is children's Grade Point Average (GPA) – reflecting an average of grades for several subjects—at age 16, namely at the end of compulsory school. To enhance comparability of the GPA scores across the cohorts analysed, we standardise the scores within each graduation year, resulting in a standardised variable with a mean of 0 and a standard deviation of 1.

### 2.4 Maternal Education

We stratified the analyses by the mother's education, which we measure by October 1st of the year in which the child was born, through a binary variable: primary or secondary (lower level) and tertiary level (higher level). We chose to focus on maternal education rather than parental education for three reasons. First, not all children have a father in the household. Second, maternal education is a strong predictor of childbearing postponement and, therefore, of the use of ART. Finally, maternal education is highly correlated with father's education, and combining the two is unlikely to result in substantially different levels of education.

### 2.5 Covariates

We controlled for a set of baseline characteristics: the child's sex (boy or girl), birth cohort (1‐year categories, 1985–2002), and plurality (singleton, twin, triplet, or higher). We also controlled for covariates that are likely to confound the association between the mode of conception and school scores: the birth order (first birth, second birth, third, fourth, fifth or higher order birth), maternal age at birth (categorical; ≤ 18, 19, 20 …. 41, ≥ 42), mean annual parental (maternal and paternal combined) income in NOK (missing, 0, 1–49,999, 50,000‐99,999, …. 750,000‐799,999, ≥ 800,000; measured two years before the birth to capture a time point before the start of the pregnancy seeking process which might lower family income for couples who undergo ART) and family structure at the beginning of the calendar year before the child's birth (parents are married/cohabiting or not living together). ART‐conceived children tend to be more likely to be firstborn, to have older mothers, to grow up in more advantaged families and stable family structures [8]–factors which can be associated with school performance [14, 15, 16, 17]. Finally, in an additional model, we accounted for two birth outcomes that might act as mediators between ART conception and school scores: whether the child was born with low birth weight (LBW, weighing less than 2.5 kg) or preterm (< 37 weeks of gestation).

### 2.6 Exclusions

From births in Norway included in the Medical Birth Register in the relevant birth cohorts, we excluded those who no longer lived in Norway at age 16 (2.6%), those without a GPA score (3.3% i.e., students for whom there wasn't a grade in more than 50% of the subjects), those whose father was not identified (0.7%), and those who had either their mother or father not resident in Norway when the child was 16 (3.2%).

### 2.7 Statistical Analysis

We estimated seven models for each parental education group to see whether the link between being ART‐conceived and school grades at age 16 is negative or positive, whether it varies between higher and lower educated parents, and whether it is explained by the sociodemographic factors mentioned above, and to the same extent in both educational categories. More specifically, Model 1 is the baseline association between being ART‐conceived (as opposed to spontaneously conceived) and school scores and includes only adjustment for basic confounders (child's sex, birth cohort and plurality); Models 2–5 adjust for the basic confounders and, separately, for the child's birth order (Model 2), maternal age at birth (Model 3), parental income two years before the child's birth (Model 4) and family structure at age 15 (Model 5), which enables us to explore the factors underlying the ART‐educational outcomes association for higher and lower educated groups. Model 6 includes adjustments for all variables (separately included in Models 2–5). Our main model specifications are 1–6.

We ran additional analyses, which we present as Supporting Information. First, we estimated Model 6 with an additional adjustment for the child's birth outcomes (low birth weight and preterm birth). Second, we estimated predicted values based on Model 1 and Model 6 by education.

### 2.8 Missing Data

The level of missingness was low, given that the data were extracted from administrative registers. The proportion missing for income was 3.3%. In the analyses we created a separate income category for those with missing income and thus these cases were retained in the analyses (in the analytical sub‐sample, the % with missing income ranged from 0.1% to 3.3% across groups depending on educational level and whether ART was used, see Table 1). Parental education was missing for 1.5% of cases and there was no missingness for family structure and for any of the other variables. Some cases fell into more than one of these categories and the total level of missingness was 4.7%.

**TABLE 1:** Sample descriptive characteristics, by parental education and mode of conception.

	Primary/secondary educated		Tertiary educated	
	SC	ART	SC	ART
% Girl	48.9	48.1	48.7	49.3
% Plurality	2.2	38.7	2.6	38.7
Average birth cohort	1993	1997	1995	1997
% First order birth	41.3	68.2	42.7	72.5
Mean maternal age	27.6	32.5	30.8	33.9
% Missing annual income	3.1	0.1	2.3	0.1
Mean annual parental income (1000 NOK)	203	327	298	459
% Parents living together	63.0	95.4	75.4	95.1
% Low birth weight	4.3	25.9	3.5	20.4
% Preterm	13.9	43.8	10.6	40.8
Number of observations	647,347	5640	272,570	3460

## 3 Results

Table 1 shows the descriptive characteristics of the analytical subsamples. As expected, a larger proportion of children are conceived via ART in the higher‐educated group than in the other educational category. Within each educational group, women who conceived via ART tend to be older than their counterparts who conceived spontaneously; they have higher parental income levels and more often live with the father of the child. Also, the well‐known relationship between ART and birth order appears. All this should be expected to contribute to better educational achievements among ART children. In contrast, in the case of ART it is more likely that the birth was a multiple birth and that the child had low birth weight and/or was born preterm, which likely contributes in the opposite direction.

The relationships between ART and these sociodemographic factors are not equally strong in the two educational categories, however. For example, the primary/secondary educated who conceived via ART are on average 6 years older than their counterparts who conceived spontaneously, whilst the corresponding difference for the tertiary educated group is 4 years. Family stability is also more closely related to ART among the less educated than the better educated, as is parental income, but only on a relative scale. However, this does not necessarily mean that these factors contribute more to the association between ART and school performances among the less educated; that depends on how the importance of these factors for the school performances varies with the mother's education.

Figure 1 shows the ART coefficients from the models regressing GPA scores on ART (coefficients displayed in Table S1). Baseline Model 1 shows that being ART conceived is positively associated with GPA scores in both educational groups. However, the association is larger in the lower‐educated group (0.17, 95% CI 0.14, 0.19) than in the higher‐educated group (0.06, 95% CI 0.03, 0.09).

**FIGURE 1:**
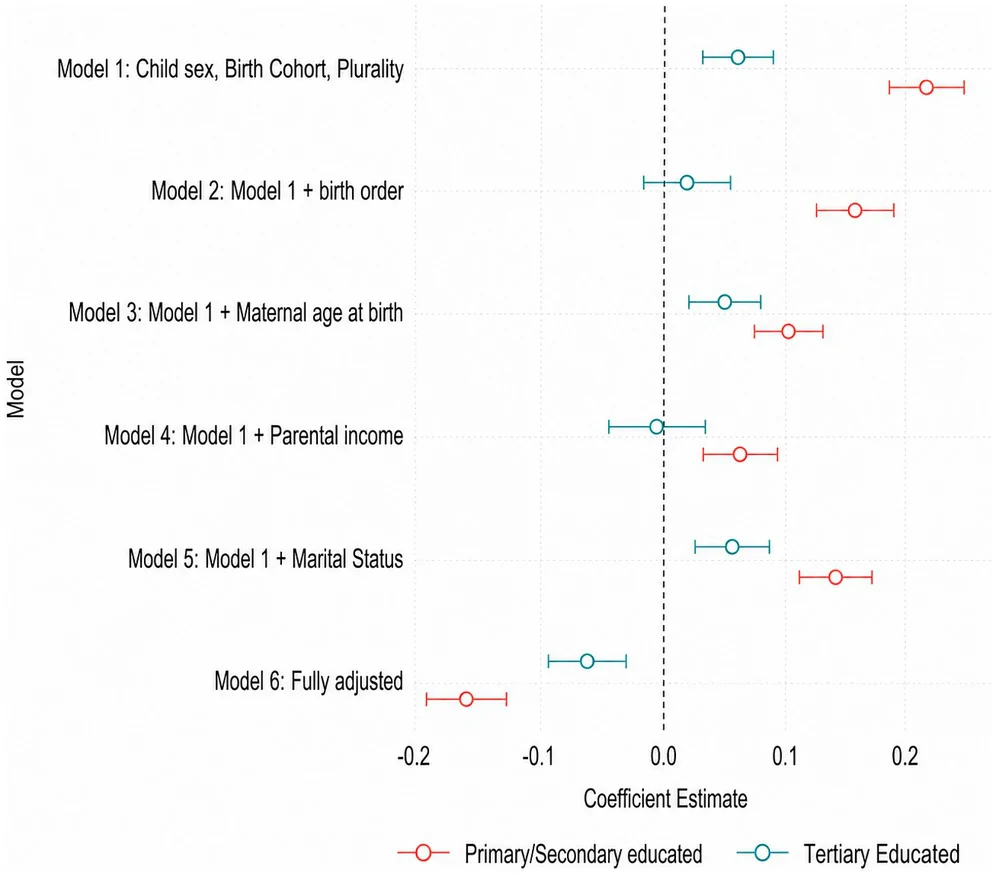
ART coefficients across Models 1–6, by maternal education.

The associations are attenuated with adjustment for covariates in Models 2–6. In the lower educated group, adjustment for income results in the largest attenuation compared to Model 1 results (70%) followed by maternal age at birth (47%), parental family structure (34%), and birth order (23%). In the higher educated group, adjustment for income results in the largest attenuation (101%), followed by birth order (60%), parental family structure (16%), and maternal age (11%). In Model 6 when all the covariates are adjusted for, the association is reversed and becomes negative in both education groups but larger in the primary/secondary (−0.18, 95% CI −0.21, −0.16) than tertiary (−0.08, 95% CI −0.11, −0.05).

We ran two additional analyses, shown in the supplementary analyses section. Table S1 shows that adjustment for birth outcomes results in only minor changes in the association compared to the fully adjusted Model 6 results. Table S2 presents predicted GPA scores by education in Model 1 and 6 and shows that both ART and spontaneously conceived children of less educated mothers have lower school scores than children of tertiary‐educated parents, in both baseline (Model 1; Table S2) and fully adjusted models (Model 6; Table S2).

## 4 Comment

### 4.1 Principal Findings

Three main findings emerged from the results. First, the results show that ART‐conceived children have higher school grades than spontaneously conceived peers in unadjusted models, particularly among those with less educated mothers. This pattern reflects both stronger socio‐demographic selection among lower‐educated parents who use ART—especially regarding maternal age and relationship stability—and differences between the two education groups in how these correlates of ART relate to children's school performance. Second, the association between ART and school grades was more strongly attenuated by controls for maternal age, parental income, and relationship stability among the less educated group than among the more educated, although the underlying factors appear broadly similar across groups. Third, the association turned slightly negative in fully adjusted models, with a somewhat larger negative coefficient among the less educated.

### 4.2 Strengths of the Study

A major strength of this study is the use of nationwide Norwegian register data covering all live births between 1985 and 2002, resulting in a large sample size of ART‐conceived children. The linkage of population, birth, education, and income registers enabled prospective analyses with minimal attrition and low levels of missing data. The study also benefited from detailed information on socio‐demographic characteristics and educational outcomes, allowing us to examine how the association between ART and school performance differs by maternal education and to assess the contribution of multiple family characteristics through sequential adjustment models.

### 4.3 Limitations of the Data

We were unable to tease out the explanations underlying the relatively small negative net effect of ART (which is unlikely to be cause of concerns) mentioned above. Second, we distinguish couples based on whether they have conceived via ART or spontaneously, but we do not observe the full infertility journey. For example, we do not know the age when the couple started seeking medical treatments for infertility, how many ART cycles the couple underwent, as well as whether they underwent less invasive MAR treatments (e.g., artificial insemination) before resorting to ART. As a result, we are unable to distinguish couples based on the severity of their infertility and their ART journey. This is a limitation because lower educated women may experience different infertility trajectories—such as seeking treatment earlier or facing more severe infertility—and more obstacles in accessing ART, which could translate into greater stress and potentially contribute to educational differences in the net effect of ART. Finally, we were unable to distinguish frozen from fresh embryo transfers, though, based on the previous studies [18], we do not expect this factor to make a difference in terms of education of ART conceived children.

### 4.4 Interpretation

A few hypotheses can be put forward as to why ART might be negatively associated with school performance in the most complex models. The ART technology per se might result in epigenetic modifications that might influence development, but evidence is not conclusive [19, 20]. The underlying infertility could be associated with genetic or biological conditions that affect the child's cognitive development, but it is unlikely that this would play a major role [21]. Couples who undergo ART might struggle in terms of mental health due to infertility, which can carry a negative effect on other outcomes such as their children's school performance [22]. Although these results are unlikely to be a cause of concern or to be linked to the (MAR) treatments per se, it is important to acknowledge that if we observed more about these families (such as strong desires for a child which are protective towards children's outcomes), the net association might become more negative.

Taken together, our findings provide little evidence that ART amplifies educational inequalities. While children conceived via ART to less educated mothers do not perform as well as those of tertiary‐educated parents, their school achievements are similar to or slightly better than those of spontaneously conceived peers from comparable educational backgrounds before adjustment, and only marginally lower once family characteristics are accounted for. Indeed, these negative differences in the fully adjusted models were quite small in magnitude: they were smaller than the positive associations estimated in the baseline models, and small compared to, for example, the 0.5 coefficient for being a girl (results not shown but available on request). This indicates that ART use among less educated parents does not create a “double disadvantage.” Rather, the educational outcomes of ART‐conceived children largely mirror the socio‐economic resources and family contexts of their parents. This interpretation is supported by supplementary analyses showing that, in absolute terms, both ART and spontaneously conceived children of less educated mothers have lower school scores than children of tertiary‐educated parents.

## 5 Conclusions

The findings highlight the importance of considering social stratification in research on ART outcomes. As ART becomes more accessible to and increasingly utilised by socio‐economically diverse groups, understanding how family resources shape the life chances of ART‐conceived children will be increasingly important for scholars, clinicians, and couples considering or undergoing fertility treatments.

## Author Contributions

A.G. came up with the idea. Both authors designed the study. Ø.K. ran the analyses. Both authors interpreted the results. A.G. wrote the first draft with support from Ø.K. Both authors revised the manuscript and approved the final draft.

## Funding

Alice Goisis acknowledges funding from the European Union's Horizon 2020 research and innovation program under grant agreement No. 803958 (MARTE). Øystein Kravdal's work has been co‐funded by the European Union (ERC, BIOSFER, 101071773). Views and opinions expressed are, however, those of the authors only and do not necessarily reflect those of the European Union or the European Research Council. Neither the European Union nor the granting authority can be held responsible for them. The work has also received funding from the Research Council of Norway through the Centres of Excellence funding scheme (project number 262700).

## Ethics Statement

Ethics approval for the use of the data was obtained from the Regional Committees for Medical and Health Research Ethics (approval # 2018/434).

## Conflicts of Interest

The authors declare no conflicts of interest.

## Supporting information


**Table S1:** ART coefficients from Models on GPA school grades, by parental education
**Table S2:** Predicted GPA scores at age 16 (with 95% CI), from Model 1 and 6 (by mode of conception and education). Except for mode of conception, all covariates are set at their mean level.

## Data Availability

The data used in this study are reckoned as highly sensitive. They can only be used in collaboration with the Centre for Fertility and Health at the Norwegian Institute of Public Health.
