# Supplementary material for: Assisted Reproductive Technologies and Offspring's School Grades by Maternal Education
Source: Paediatr Perinat Epidemiol. 2026 Jul 16;40(7):805–10. doi: 10.1111/ppe.70181 (PMC13624520; doi:10.1111/ppe.70181)
Supplement: Supplementary file 1 [file PPE-40-805-s001.docx]

| **Table S1 ART coefficients from Models on GPA school grades, by parental education** | | | | | | | |
| --- | --- | --- | --- | --- | --- | --- | --- |
|  | **Tertiary educated** | | |  | **Primary/Secondary educated** | | |
|  | **Coeff** | **95% CI** | |  | **Coeff** | **95% CI** | |
| Model 0 | 0.0244 | -0.0036 | 0.0523 |  | 0.1499 | 0.1243 | 0.1754 |
| Model 1: Child sex, Birth Cohort, Plurality | 0.06 | 0.03 | 0.09 |  | 0.17 | 0.14 | 0.19 |
| Model 2: Model 1 + Birth order | 0.02 | -0.01 | 0.05 |  | 0.13 | 0.10 | 0.16 |
| Model 3: Model 1 + Maternal age at birth | 0.05 | 0.02 | 0.08 |  | 0.09 | 0.06 | 0.12 |
| Model 4: Model 1 + Parental income | 0.00 | -0.03 | 0.03 |  | 0.05 | 0.03 | 0.08 |
| Model 5: Model 1 + Marital Status | 0.05 | 0.02 | 0.08 |  | 0.11 | 0.09 | 0.14 |
| Model 6: Fully adjusted | -0.08 | -0.11 | -0.05 |  | -0.18 | -0.21 | -0.16 |
| Model 6 + LBW + Preterm | -0.08 | -0.10 | -0.05 |  | -0.17 | -0.19 | -0.14 |

| **Table S2 Predicted GPA scores at age 16 (with 95% CI), from Model 1 and 6 (by mode of conception and education). Except for mode of conception, all covariates are set at their mean level.** | | | | |
| --- | --- | --- | --- | --- |
|  | **Primary/Secondary educated** | | **Tertiary Educated** | |
|  | SC | ART | SC | ART |
| Model 1 | -0.20 (–0.20 to –0.19) | -0.033(–0.06 to –0.07) | 0.533 (0.53 to 0.54) | 0.59 (0.56 to 0.62) |
| Model 6 | -0.20 (-0.20 to –0.20) | -0.38 (-0.41 to -0.36) | 0.53 (0.53 to 0.54) | 0.45 (0.43 to 0.48) |
